# A Brief Report: Lessons Learned and Preliminary Findings of Progreso en Salud, an HIV Risk Reduction Intervention for Latina Seasonal Farmworkers

**DOI:** 10.3390/ijerph14010032

**Published:** 2016-12-30

**Authors:** Mariano Kanamori, Mario De La Rosa, Stephanie Diez, Jessica Weissman, Mary Jo Trepka, Alicia Sneij, Peter Schmidt, Patria Rojas

**Affiliations:** 1 Center for Research on US Latino HIV/AIDS and Drug Abuse, Florida International University, Miami, FL 33199, USA; delarosa@fiu.edu (M.D.L.R.); sdiez002@fiu.edu (S.D.); jweis005@fiu.edu (J.W.); trepkam@fiu.edu (M.J.T.); asnei001@fiu.edu (A.S.); proja003@fiu.edu (P.R.); 2 School of Social Work, Robert Stempel College of Public Health & Social Work, Florida International University, Miami, FL 33199, USA; 3 Department of Health Promotion and Disease Prevention, Robert Stempel College of Public Health & Social Work, Florida International University, Miami, FL 33199, USA; 4 Department of Epidemiology, Robert Stempel College of Public Health & Social Work, Florida International University, Miami, FL 33199, USA; 5 Department of Political Science, University of Gießen, Gießen 35390, Germany; Peter.Schmidt@sowi.uni-giessen.de; 6 Department of Psychology, Cardinal Stefan Wyszyński University, Warszawa 01-815, Poland

**Keywords:** hispanic Americans, farmworkers, HIV, primary prevention

## Abstract

Throughout the past decade, HIV rates in Florida—particularly South Florida, where many Latina seasonal farmworkers reside and work—have ranked among the highest in the nation. In this brief report, we delineate important lessons learned and preliminary findings from the implementation of the HIV prevention intervention *Progreso en Salud* (Progress in Health). Among the 114 Latina seasonal farmworker participants, there were significant increases from baseline to 6-month follow-up in the percentages of overall condom use, HIV testing, HIV/AIDS-related communications with friends, HIV knowledge, condom use self-efficacy, and correct use of condoms. Lessons learned from this study can be used to inform future HIV intervention strategies to improve the adoption and maintenance of HIV risk reduction behaviors among high-risk Latina seasonal workers and other high-risk underserved populations. Future research is needed to support our findings.

## 1. Introduction

Agriculture is a major industry in Florida [1]. In Miami-Dade County, South Florida, the agricultural industry generates more than $2.7 billion in profit each year [2]. Miami-Dade produces 99% of the state’s sweet potatoes, 64% of the squash, and 35% of the beans [3]. The plant nursery industry in Miami-Dade County is also large and diverse. Miami-Dade County ranks first in Florida and second in the nation in ornamental plant production. Due to Florida’s strong agricultural industry and proximity to Latin America, the state is a magnet for Latino/a seasonal workers. Between 150,000 and 200,000 migrant and seasonal farm workers work in Florida every year [4]. Of those, more than 20,000 migrant and seasonal workers live in Miami-Dade County [2].

Latina female seasonal farmworkers (Latina seasonal workers) in the United States are an underserved population, with approximately 75% living below the poverty level [5] and with average total family income ranging between $17,500 and $19,000 [6]. In the present study, we define Latina seasonal worker to be a Latina who works or whose spouse/partner works in agriculture at a single location within 75 miles of their home throughout the year [7]. The marginalization that Latina seasonal workers experience, including limited access to health care and poor health conditions, is reflected in their average life expectancy of 49 years [8] and the scarce number of studies investigating effective interventions to improve their health and wellbeing.

In the US, the 2015 HIV diagnosis rate was three times higher for Latinos (16.4 per 100,000) than for non-Latino Whites (5.3 per 100,000) [9]. In 2015, Florida had the third highest HIV diagnosis rate in the United States (24.0 per 100,000) after the District of Columbia (57.0 per 100,000) and Louisiana (24.2 per 100,000) [9]. In 2014, Florida’s Latino residents had a 55% higher HIV diagnosis rate (37.6 per 100,000) than the national rate for Latinos (24.2 per 100,000) [10]. Within Florida, in 2015, the HIV diagnosis rate was nearly three times higher among Latinos (30.8 per 100,000) than among non-Latino Whites (10.9 per 100,000) [11]. In 2015, the HIV diagnosis rate in the South Florida region, where many Latina seasonal farmworkers reside and work, was the highest among all metropolitan areas of the country (38.8 per 100,000) [9]. While there is a lack of specific data on the prevalence of HIV among Latina seasonal workers living and working in Miami-Dade County, few studies have examined the HIV risk behaviors of this population. A study that interviewed 278 Latina seasonal workers in Miami-Dade County found that due to cultural and gender roles, HIV prevention is not often discussed among Latinos [12], which is concerning given that HIV disproportionately affects the Latino population.

The incorporation of social and cultural context into evidence-based interventions has been identified by HIV researchers as crucial for improving access and quality of care for racial/ethnic minorities [13]. In this context, the Centers for Disease Control and Prevention (CDC) has promoted the implementation of evidence-based behavioral interventions in their HIV prevention programs [14]. To date, research has supported the development and evaluation of culturally-competent HIV prevention programs for Latinas that address the specific needs of this population. One such example is the project *AMIGAS* (which means “friends” in Spanish), a culturally congruent HIV prevention intervention for Latina women adapted from *SiSTA*, an intervention for African American women [15,16]. *AMIGAS* demonstrated marked increases in HIV-protective behaviors for Latina women living in a metropolitan area. Another example is *Salud, Educación*, *Prevención*, *y Autocuidado* (*SEPA*, Health, Education, Prevention, and Self-Care) [17]. The SEPA intervention consists of three sessions covering STI and HIV prevention; communication, condom negotiation, and condom use; and violence prevention. Results from the SEPA intervention indicated moderate efficacy on improved condom use, improved communication with partner, improved HIV-related knowledge, and decreased barriers to condom use. Other culturally tailored evidence-based HIV prevention interventions with a sample including only a proportion of Latinas have not solely been tailored to the Latino culture [18,19,20,21]. Moreover, most culturally tailored evidence-based HIV prevention interventions with Latinas have focused on adolescents [22,23,24,25,26,27,28,29]. Other evidence-based HIV prevention interventions simultaneously included males and females from different racial/ethnic populations. One example is Video Opportunities for Innovative Condom Education and Safer Sex (VOICES/VOCES), a single-session HIV/STD prevention intervention that emphasizes condom use and condom negotiation skills among African American and Hispanic men and women. VOICES/VOCES has been adapted to specific populations such as individuals in correctional settings and men who have sex with men [30] but has not been previously adapted to Latina seasonal workers. A description of VOICES/VOCES is included in the Methods section. A study by Fisher et al. with data from four CDC-funded community agencies implementing VOICES/VOCES (N = 922 participants from different racial/ethnic groups) found significant risk reductions at 30- and 120-days post-intervention for all outcome measures (e.g., unprotected sex) [30]. Furthermore, Fisher et al. found that, for reducing number of individuals who have multiple sex partners and frequency of unprotected sex, VOICES/VOCES had greater effect among Latinos compared to Whites [30].

The lack of culturally tailored evidence-based HIV prevention programs for Latina seasonal workers led to the development and implementation of a community based participatory HIV prevention program, titled “HIV Risk Reduction in Latina Seasonal Workers in South Florida.” Researchers have recognized the importance of adapting the delivery and content of an intervention so that communities could develop a sense of ownership over the intervention [31]. It has been suggested that the adaptation of evidence-based interventions should be the rule rather than the exception [32]. When we presented the original version of VOICES/VOCES to our community partner agency, they requested an adaptation of the intervention that incorporates the realities faced by the community and its members. In conjunction with our community partner, we culturally tailored the evidence-based intervention that was later pre-tested in the community.

This HIV prevention intervention program is based on a two-group randomized design approach, with the *Progreso en Salud* (Progress in Health) intervention as the test condition and a health promotion intervention as the control. Currently, 6- and 12-month follow-up is being conducted to determine the efficacy of both the test and control condition of this study. In the present brief report, we summarize our lessons learned and present preliminary findings related to the reduction of risky sexual behaviors and the increase of condom use in this population. These preliminary results are from data collected on 114 Latina seasonal workers who attended the Progreso en Salud intervention. All 114 participants completed the baseline and 6-month follow-up interviews. As this study is in-progress, complete 6-month follow-up data from participants assigned to the health promotion intervention were not yet available.

## 2. Materials and Methods

### 2.1. Study Sample

Our convenience non-clinical sample consisted of 114 at-risk Latinas who met the following eligibility criteria: living in a seasonal farmworker camp; being 18 years of age or older; able to speak and understand Spanish; reporting at least one episode of unprotected sex and consumption of alcohol or other drugs in the 3 months prior to baseline interview; willingness to be randomized to intervention or control group and to be contacted by phone or home visits for the follow-up interviews; likely to remain in the general geographic area for the next 2 years; and, able to understand and provide written informed consent.

Sample demographics, presented in Table 1, indicate that the highest proportion of participants were between the ages of 30–39 (42.9%) followed by 40–49 (23.2%) years, and the majority of our sample was either legally married (42.0%) or cohabitating (25.0%). Nearly one quarter of the participants were single (24.1%). The highest level of education ranged from high school diploma (8.0%) or some undergraduate education (8.0%) to 1st–8th grade (43.8%). Most participants were born in Mexico (65.0%), followed by the United States (18.0%) and Guatemala (9.0%), and they reported living in their current community for an average of 13.6 years. Total income during the past 30 days ranged from less than $50 (3.6%) to $600 or more (30.6%). This was a largely medically uninsured sample, as almost three out of every four participants (76.8%) did not have health insurance.

### 2.2. The Progreso en Salud Intervention

The intervention, Progreso en Salud, is an adaptation of the evidence-based VOICES/VOCES intervention. VOICES/VOCES is a video-based intervention that is grounded in the Theory of Reasoned Action. According to the Theory of Reasoned Action, individuals’ behaviors are guided by two factors: (1) their attitudes, beliefs, and experiences; and (2) how they believe others think they should act in a given circumstance (i.e., the social and cultural norms of their community). VOICES/VOCES is one of the research-based interventions identified by the Diffusion of Effective Behavioral Interventions Project (DEBI), a project initiated by the CDC to help bridge the gap between HIV/STD prevention research and practice [33]. DEBI identifies HIV/AIDS prevention interventions with demonstrated evidence of effectiveness and supports the original researchers in developing a user-friendly package of materials designed for health promotion professionals [34].

The original VOICES/VOCES is a single 45-min session, video-based program for the prevention of HIV and other sexually transmitted diseases. VOICES/VOCES was designed to encourage condom use and improve condom negotiation skills among heterosexual African American and Latino men and women, aged 18 years and older, who are at very high risk for HIV and other sexually transmitted diseases [35]. Table 2 presents the four core components of VOICES/VOCES and the corresponding adaptations made for the present study.

### 2.3. Pre-Implementation of Progreso en Salud

The pre-implementation phases included the following activities: revising the intervention kit materials; assessing the available resources and cost of implementation; developing the program budget; identifying appropriate setting, space, and equipment with the collaborating community partner; assessing staff’s capacity to conduct the intervention; training staff members using the VOICES/VOCES manual on leading skill-building sessions, condom use and negotiation skills, and evaluation tools for program implementation; identifying and developing adequate and innovative methods to recruit Latina seasonal workers at risk for HIV; identifying and hiring members from the community with adequate skills to perform small group facilitation and recruitment of participants; implementing the human subjects protection training with all personal involved in the program; establishing recruitment partnerships with over 30 local/state health officials and private and public organizations working with the seasonal worker community; implementing a qualitative study (3 focus groups; N = 29 Latina seasonal workers) to gain specific information regarding this community; and developing an adaptation of the intervention implementation using a community-based participatory research approach. Our partnering community-based organization played a key role in all phases of the study, including delivery of the intervention and data collection. Study participants were recruited at locations where seasonal farmworkers are known to reside, including trailer parks, dormitory-style housing, apartment buildings, motels, duplexes, and neighborhoods of single/duplex housing, or farm work places in the Homestead/Florida City area (Miami-Dade County).

### 2.4. Adaptation of VOICES/VOCES

The strategy of the original VOICES/VOCES included the recruitment of participants among patients attending sexually transmitted disease/infection clinics. Although the original VOICES/VOCES was implemented in clinics specializing in sexually transmitted diseases, Progreso en Salud was conducted in the private, safe offices of a community-based organization—located inside the Latino seasonal community in Homestead/Florida City. Table 2 presents the four core components of VOICES/VOCES and the adapted four components of Progreso en Salud.

Our adapted recruitment strategy was based on a respondent-driven sampling approach. Our Project Coordinator and Lay Health Advisor invited 10 Latina Leaders in the seasonal worker community to participate in the study and commence the respondent-driven recruitment. Members from community-based organizations identified the leaders—respected women in the community who were able to reach a large number of peers. Each of the 10 Latina Leaders invited three friends, and these friends invited three additional participants who were willing and eligible to participate in the study, until a total of 13 women (including the Latina Leader) were recruited into a group. Participants were informed that these friends needed to be women with whom they discuss important issues such as health, family planning, and HIV/AIDS.

The Progreso en Salud intervention session lasted approximately three hours. In our pre-testing of the intervention session, we found that 45 min was not sufficient time to complete all the activities proposed in the original VOICES/VOCES. Latina seasonal workers were able to attend the intervention session at lunch or dinner times. As such, we implemented the interventions around noon or early evening and offered lunch/dinner.

Participant incentives included a lunchbox and $40 for completing the baseline interview, $40 for attending the Progreso en Salud intervention, and $50 for completing the 6-month follow-up interview. The Latina Leader also received $10 for each eligible participant that was recruited into her group, $10 for each member of her group who attended the Progreso en Salud intervention, and $10 for each friend with whom she discussed the informational materials three months after the Progreso en Salud intervention. The interventions were audio-recorded to assess the fidelity of the implementation. This study was approved by Florida International University’s institutional review board (IRB-14-0352).

### 2.5. Baseline Interviews

At baseline, participants completed a structured individual face-to-face interview (approximately 1.5 h in length) in Spanish using computer assisted personal interviewing (CAPI) software in a private office of the collaborating community agency. Interviews were performed by six bilingual Latinas. All measurements have been tested and validated with minority populations and have primarily been used with Latinas in South Florida. The questionnaire, originally developed in English, was translated into Spanish and back-translated to English to ensure the integrity of the instrument and language translation competencies. The instrument was pilot tested twice with a total of 10 participants to confirm that the content and language would be clear to participants. Trained bilingual interviewers provided written informed consent forms to eligible participants. Data quality control was performed by a PhD-level scientist. Our Lay Health Advisor performed retention activities via phone calls and home visits. The Social and Behavioral Institutional Review Board of Florida International University approved this study (IRB-14-0352).

*Condom use* was assessed with four items regarding the frequency of condom use during vaginal and anal sex in the past 30 days, with (a) primary sex partner and/or (b) casual sexual partner (e.g., “How often was a condom used for anal sex with your primary sex partner during the past 30 days?”). Questions were measured on a scale from 1 (“Always”) to 4 (“Never”). The 4 questions about frequency of condom use (i.e., vaginal sex with primary partner, vaginal sex with casual partner, anal sex with primary partner, anal sex with casual partner) were combined into a single variable of condom use that classified a person according to the least frequent condom use. For example, if the participant reported never using a condom for vaginal or anal sex with a primary sex partner and sometimes using a condom for vaginal or anal sex with a casual sex partner, that person’s condom use was coded as “never”.

*HIV testing* was assessed by asking, “Have you been tested for HIV during the past 6 months?”

*The frequency of communication with friends regarding HIV prevention* was assessed with the following two questions: “Have you talked with your friends about ways to convince your partner to use condoms?” and “Have you talked with your friends about ways to make sex with a condom fun?” Response options on a 5-point scale included “very often”, “often”, “sometimes”, “not often”, and “never”.

*HIV-related knowledge* was assessed with 18 true/false items from the HIV Knowledge Questionnaire (HIV/-KQ-18) (e.g., “coughing and sneezing do not spread HIV”) [36]. The total score indicated the number of items answered correctly, with 1 point per each correct answer. The total score ranged from 0–18. A higher score indicated a higher level of HIV-related knowledge.

*Participants’ condom use self-efficacy* was assessed with a seven item, 4-point Likert-type scale with scores ranging from 1 (“strongly disagree”) to 4 (“strongly agree”). The seven items were: *If I decide to use condoms, I can have (all) my partner(s) use them*; *It would be easy to make my partner use a condom*; *I have enough self-control to use condoms whenever I have sex*; *It is difficult to tell my partner “no” to sex without a condom*; *If I am in a sexual relationship, there is little I can do*; *I am sure I can put a condom on my partner without it breaking or falling off*; and, *I feel comfortable speaking to people in my community about HIV/AIDS* [37,38]. The score from items stating ideas that did not reflect positive condom use self-efficacy were reversed (i.e., *It is difficult to tell my partner “no” to sex without a condom*). The total score was calculated by summing the scores from the seven items (range = 7–28) with a higher score representing a higher level of self-efficacy for HIV prevention.

*Correct use of condoms* was measured using a five item, 5-point Likert-type scale with scores ranging from 0 (“never”) to 4 (“very often”). The measure included the following items: *In the past, when you wanted to use a condom, you have been able to use it correctly*; *You have used condoms without them breaking*; *You have used condoms without them leaking*; *You remember to hold the base of the condom when you take it off*; and, *You remember to check the expiration date before using a condom*. The total score was calculated by summing the scores from the five items (range = 0–20), with a higher score representing a higher level of correct use of condoms.

### 2.6. Six-Month Follow-Up Interview

Six months after receiving the Progreso en Salud intervention, participants completed a structured face-to-face individual interview (approximately 1.5 h in length) using computer assisted personal interviewing software (CAPI) in a private office of a community-based organization. The same questionnaire used during baseline interviews was implemented at 6-month follow-up.

### 2.7. Data Analysis

Descriptive statistics at baseline included proportions for categorical variables and means and standard deviations for continuous variables. Knowledge of partner’s HIV status and HIV testing were assessed using McNemar’s test. Related Samples Marginal Homogeneity Test was performed to assess significant changes in the proportion of women who used condoms when having sex (vaginal and/or anal) with primary sex partner and/or sex (vaginal and/or anal) with a casual sexual partner in the past 30 days, and to assess significant changes in the proportion of women who talked to their friends about ways to convince their partner to use condoms and ways to make sex with a condom fun. Related Samples Wilcoxon Signed Rank Test was used to analyze changes in HIV knowledge, condom use self-efficacy, and correct use of condoms. Significant associations were determined with *p* values (0.05) and 95% confidence intervals (CIs). Effect sizes were calculated by dividing the Z score by the square root of the number of observations (baseline and 6-month follow-up). Missing data was addressed using pairwise deletion.

## 3. Results

*Condom Use.* Significant differences from baseline to 6-month follow-up were found in the percentages of overall condom use (*p* = 0.046) (Table 3). A higher percentage of women at 6-month follow-up used condoms during the past 30 days every time (13.5% vs. 9.6%) or sometimes (24.0% vs. 17.3%) when they had vaginal and/or anal sex. There was also a decrease in the percentage of women who never used condoms (57.7% vs. 49.0%) when they had sex.

*HIV Testing.* There was a significant increase in HIV testing from baseline (15.0%) to 6-month follow-up (27.9%) (*p* = 0.019) (Table 3). Although knowledge of partner’s HIV status increased by 7.2 percentage points from 34.4% to 41.6%, this increase was not statistically significant.

*HIV/AIDS Related Communications with Friends.* There was a significant change in the distribution of the proportions of women who planned to talk to their friends about ways to convince their partner to use condoms more frequently (*p* < 0.001) (Table 3). For example, there was an increase in the percentage of participants who very often had those discussions (0.0% to 15.0%) and a decrease who reported that they would never have those discussions (54.1% to 30.0%). Similarly, women were more likely to report more frequently talking with fiends about ways to make sex with a condom fun (*p* < 0.001). For example, the percentage who reported having those discussions very often increased (from 2.5% to 6.1%) and the percentage that reported never having those discussions decreased (from 76.2% to 44.4%).

*HIV Knowledge.* Prior to the program, the mean of the HIV knowledge score was 9.43 and six months after the program, the mean score was 11.33, representing a statistically significant mean increase from baseline to 6-month follow-up (*p* = 0.001), with a relatively large effect size (r = 0.46) (Table 3).

*Participants’ Condom Use Self-Efficacy*. The mean score of condom use self-efficacy increased from 21.25 to 22.21 (*p* = 0.005), with a small effect size (r = 0.21), (Table 4).

*Correct Use of Condoms.* Participants’ correct use of condoms mean scores increased from 9.36 to 10.64, (*p* = 0.005), with a small effect size (r = 0.20), (Table 4).

## 4. Discussion

The findings from this study suggest that the Progreso en Salud intervention may have helped increase study participants’ knowledge about HIV and practices regarding condom use and HIV testing. Our results also suggest that many participants could be in the process of forming their condom use self-efficacy as a result of the intervention. Future research is needed to support our findings.

Our results suggest that existing best practice interventions such as VOICES/VOCES, which have been widely used to address health care concerns affecting Latinas, can be tailored to improve HIV knowledge, HIV testing, and condom use among Latina seasonal workers. For such interventions to be successful, they must address Latino cultural values and should be conducted in an environment that allows Latina seasonal workers to feel comfortable discussing these sensitive topics. We implemented the intervention in a community-based organization because members from this community expressed that many Latina seasonal workers many not feel comfortable attending sexually transmitted disease clinics. Members from the community also mentioned that attending the session at an STD clinic could potentially trigger stress or anxiety in some participants, who may have been concerned that friends or partners might believe that they were in need of STD services—if seen around or inside these clinics.

The Latino cultural value of *personalismo* refers to a preference by Latinos for friendships with individuals that are affiliated with their social groups—suggesting a preference for familiarity in these relationships [39]. Latinos are more likely to cooperate with and trust someone with whom they have had pleasant conversations. Compared to the larger American society, Latinos have a tendency to refrain from discussions of controversial issues such as HIV/AIDS because they prefer to discuss these issues after establishing a friendship/relationship based on trust, support, and caring [40]. Thus, *personalismo* was incorporated into the recruitment and configuration of the sample because Latinos prefer to hold conversations about sensitive topics, such as HIV prevention, after establishing friendship relations based on trust, support, and caring [40]. Instead of using groups of participants who did not know each other, we used a respondent-driven sampling approach in our adapted version of the VOICES/VOCES intervention, which allowed the configuration of groups with participants who were friends or who had a friend in common.

In Latino society, women who speak openly about sexuality are often viewed as promiscuous. In fact, we found that some participants were initially shy or reluctant to practice placing the condom on the penis model. One of the unique aspects of our adaptation of the original VOICES/VOCES intervention was the use of role-playing exercises in which the participants used dolls to role-play sex-related dynamics, hence increasing participants comfort and willingness to discuss sex. The participants were provided with a penis model to practice the proper procedure for condom placement, which initially made some of the participants feel hesitant to participate. However, once the more shy individuals observed their peers using the penis models to practice condom placement, they felt more comfortable and thus engaged in the activity themselves. Group members supported each other during this activity. For example, when a participant incorrectly placed a condom on the model, a fellow participant would typically show her how to properly place the condom.

*Collectivism*—close, nurturing, and supportive interpersonal relationships—is valued in most Latino cultures over individualism, which is more prominent in US culture. In many Latino cultures, it is important that individuals accept responsibility toward their families and larger community [41]. Accordingly, we included collectivist values as a major focus in our adapted version of the VOICES/VOCES intervention. Specifically, with Progreso en Salud, we provided Latina Leaders with instructions on how to promote interactions and conversations about HIV prevention within their group of friends, so that community members (Latinas) could support one another. We found that their constant interaction facilitated the development of strong connections and social capital. Participants reported being interested in the Progreso en Salud intervention not only for their personal benefit, but rather, they wanted the entire group of friends to take advantage of the intervention. For instance, some participants gave their study incentives to other participants who could not afford to pay for transportation to the intervention. In addition, several participants volunteered to host meetings in their houses or in parks to discuss HIV prevention messages. Furthermore, participants informed us that they also communicated among themselves via phone calls to remind each other about attending the intervention or scheduling an interview—and in some cases when a Latina Leader could not contact one of the participants by phone, contact was made by their friends using social media (i.e., Facebook).

Several lessons were learned relate to implementation logistics. For instance, our activities were purposefully designed to be social events—meals (lunch or dinner) were incorporated into the intervention. We promoted active participation. To allow all participants to practice correct condom placement using penis models and perform role play sex-related dynamics using the dolls, our intervention lasted three hours (vs. the 45-min-long session VOICES/VOCES intervention). Participants did not oppose or complain about staying for this length of time. Many participants expressed that it would be difficult to attend intervention sessions because they needed to care for their children. Therefore, it was crucial to offer babysitting services at no cost, which included meals for the children.

Additionally, similar to other studies with underserved populations, the use of visual materials was critical to conveying the information. Therefore, we developed and pre-tested a series of flip-chart posters with large graphical representations. These materials were used by our Project Coordinator and Lay Health Advisor to provide information and promote discussion regarding HIV/AIDS and STDs. It was important to include depictions of Latinos who had physical characteristics that resembled those of members of this Latino seasonal worker community. These pictures did not portray the stereotypical representation of Latina seasonal workers in their work clothing. Instead, the pictures were representative of the well-dressed Latinas that attended the intervention. Additionally, we updated the materials and messages included in the VOICES/VOCES kit by incorporating activities that inform and demonstrate how to use female condoms.

### 4.1. Limitations

This study has several limitations that should be noted. First, it depended on self-reported measures of sensitive information such as HIV risk behaviors, increasing the chances for social desirability bias and recall bias. However, we utilized measurements that have been previously used with other Latino populations and had existing Spanish translations that could adapted to the Latino seasonal worker population. Additionally, care was taken to utilize experienced Latina interviewers for data collection that were extensively trained in culturally appropriate interviewing techniques. Second, our intervention was conducted with a unique population of at-risk Latinas, so future studies that replicate the aforementioned methods in other regions of the United States and/or with different Latino populations may yield different results. Third, findings from this study were based on an ongoing pilot study with a small convenience sample that limits the generalizability of our findings. Fourth, data/results from the comparison group are not yet available and thus we do not know if the reported findings from the Progreso en Salud intervention led to better outcomes than the health promotion intervention. Only changes in HIV knowledge had a relatively large effect size. Fifth, the article presents only descriptive and unadjusted results for the participants (*n* = 114) receiving the intervention.

### 4.2. Future Research Orientation

Future studies should identify the specific characteristics of potentially influential individuals, the venues where they interact, and the type of seasonal work that protect or place Latina seasonal workers at risk for HIV infection. These findings could be used to tailor evidenced-based interventions for subgroups of interconnected individuals with specific demographic, cultural, or sexual behavioral characteristics including sexual abstinence or delayed onset of sexual activity, consistent condom use, and engagement in routine HIV testing for individuals who were or become sexually active. It is also important to study how changes in social networks over time place Latino seasonal workers at risk for HIV across generations. Future studies using latent growth mixture curves would allow us to assess different effects of the intervention among subgroups within the sample.

## 5. Conclusions

This paper delineates important lessons learned that can be incorporated into HIV intervention strategies to improve the adoption and maintenance of HIV risk reduction behaviors among high risk Latina seasonal workers and other underserved populations. Future research is needed to support our findings.

## Figures and Tables

**Table 1:** Sample characteristics.

Characteristics	*n* ^a^	%
Total	114	100
**Age (year)**		
18–29	23	20.5
30–39	48	42.9
40–49	24	23.2
50 and older	15	11.6
**Education**		
None	12	10.7
From 1st to 8th grade	49	43.8
9th to 11th grade	12	10.7
GED	21	18.8
Post High School Education	9	8.0
Some undergraduate education	9	8.0
**Marital Status**		
Single	27	24.1
Legally married	47	42.0
Cohabitating	28	25.0
Separated/divorced	9	8.0
Widow	1	0.9
**Country of Birth**		
Mexico	65	65.0
USA	18	18.0
Guatemala	9	9.0
El Salvador	5	5.0
Nicaragua	2	2.0
Colombia	1	1.0
Cuba	1	1.0
**Number of years living in this Latino Seasonal Worker Community (mean, SD)**	13.58 (10.98)	
**Total Income from the past 30 Days**		
No income	16	14.4
Less than $50	4	3.6
$51 to $99	6	5.4
$100 to $199	13	11.7
$200 to $399	22	19.8
$400 to $599	16	14.4
$600 or more	34	30.6
**Has health insurance**		
Yes	26	23.2
No	86	76.8

^a^ Totals may not equal 114 due to missing values.

**Table 2:** Original Core Components of VOICES/VOCES and Adapted Core Components of Progreso en Salud.

Original VOICES/VOCES	Adapted Progreso en Salud
Component 1: Present a culturally specific video portraying condom use and negotiation. The actors in the video present information on HIV/STD risk behavior and model condom use and negotiation.	We presented the novela (Latino soap opera) during lunch or dinner. While participants were eating, the Project Coordinator and Lay Health Advisor encouraged participants to discuss the scenes and various ways to handle the situations presented in the novela using a standardized protocol. These discussions followed a consistent format, but the content was tailored to address the concerns and experiences of each group. This approach was used to resemble a conversation at a social or family event.
Component 2: Condom negotiation is role-played, practiced, and discussed. Facilitators ask specific questions about the characters and events depicted in the video, and then encourage clients to relate these situations to their own lives. Facilitators provide information, correcting misinformation, discussing condom options.	Condom negotiation was role-played, practiced, and discussed after instructing participants to use dolls to enact a real-life scenario. The hypothetical scene began with the couple on a romantic date, later becoming physically intimate at his/her house. The woman suggested using a condom before proceeding with sexual intimacy and ultimately intercourse; however, the male refused to use a condom. All participants were then instructed to discuss ways to negotiate with the man to and ultimately convince him to use a condom. We included visual materials to facilitate participants’ understanding of the messages presented in the video. The Project Coordinator and Lay Health Advisor used a series of flip-chart posters with large graphical representations to provide information and promote discussion regarding HIV/AIDS and STDs including sex and drug-related modes of transmission, HIV statistics, and ways to prevent HIV/STD infection. The content of these flip-chart posters mirrored the messages included in the original VOICES/VOCES strategy. Adequate interpretation of the messages and cultural acceptance of the figures were pilot tested with members of the community. We provided Latina Leaders with additional instructions on how to promote interactions and conversations about HIV prevention within their group of friends. Three months after the intervention session, Latina Leaders met with the individuals who participated in their groups to discuss HIV/STD prevention—either as a group, in a single session, or via individual home visits depending on the participants’ availability. The Latina Leaders were given three HIV/STD informational pamphlets in Spanish to guide the conversations. These pamphlets were also distributed to each member of her group.
Component 3: Facilitators use a poster to show features of various condom brands in English and Spanish. Participants practice correct condom placement. The original VOICES/VOCES kit includes only one penis model.	Facilitators use a poster to show features of various condom brands in English and Spanish. We added information and demonstrations on how to use female condoms because the original VOICES/VOCES intervention and materials included only male condoms. Participants practiced correct condom placement. We included additional penis models in our intervention. All participants had the opportunity to simultaneously practice correct condom placement on the penis models.
Component 4: At the end of the session, participants are given samples of the types of condoms they have identified as best meeting their needs.	At the end of the session, participants were given samples of the types of condoms they identified as best meeting their needs.

**Table 3:** Results of McNemar’s Test and Related Samples Marginal Homogeneity Test of Changes in Condom Use, HIV Testing, and HIV Related Communications with Friends from Baseline to 6-Month Follow-Up.

Question	Baseline	6-Month Follow-Up	*p*-Value
	(%)	(%)	
McNemar’s Test			
**Have you been tested for HIV during the past 6 months?**			
-Yes	15.0	27.9	*p* = 0.019 *
**Do you know the HIV status of your husband or stable partner?**			
-Yes	34.4	41.6	*p* = 0.265
Related Samples Marginal Homogeneity Test			
**How often was a condom used with your sex partner during the past 30 days? ^a^**			
-Every time	9.6	13.5	*p* = 0.046 *
-Almost every time	7.7	6.3	
-Sometimes	17.3	24.0	
-Almost never	7.7	7.3	
-Never	57.7	49.0	
**Have you talk to your friends about ways to convince your partner to use condoms?**			
-Very often	0.0%	15.0%	*p* < 0.001 **
-Often	11.5%	16.0%	
-Sometimes	23.8%	33.0%	
-Not very often	4.9%	6.0%	
-Never	54.1%	30.0%	
**Have you talked to your friends about ways to make sex with a condom fun?**			
-Very often	2.5%	6.1%	*p* < 0.001 **
-Often	4.1%	14.1%	
-Sometimes	10.7%	28.3%	
-Not very often	6.6%	7.1%	
-Never	76.2%	44.4%	

^a^ Condom use was assessed with four items regarding the frequency of vaginal and anal sex with primary sex partner and vaginal or anal sex with a casual sexual partner in the past 30 days; * Significant at *p*-value < 0.05. ** Significant at *p*-value < 0.001.

**Table 4:** Related samples Wilcoxon signed rank test of changes in hiv knowledge, ability to practice hiv prevention behaviors, and correct use of condoms from baseline to 6-month follow-up.

Wilcoxon Signed Rank Test												
		Baseline					Six Month Follow-Up					
	Minimum	Maximum	Mean	Median	Std. Deviation	Minimum	Maximum	Mean	Median	Std. Deviation		r
HIV Knowledge	0.00	15.00	9.43	10.00	3.35	3.00	17.00	11.33	11.00	3.31	*p* = 0.001 ***	0.46
Condom use self-efficacy	11.00	28.00	21.25	22.00	3.62	11.00	28.00	22.21	23.00	4.01	*p* = 0.005 **	0.21
Adequate use of condoms	0.00	20.00	9.36	10.00	4.81	0.00	18.00	10.64	11.00	3.80	*p* = 0.016 *	0.20

* Significant at *p*-value < 0.05. ** Significant at *p*-value < 0.01. *** Significant at *p*-value < 0.001.
